# What is behind high turnover: a questionnaire survey of hospital nursing care workers in Shanghai, China

**DOI:** 10.1186/s12913-018-3281-9

**Published:** 2018-06-22

**Authors:** Yan Wang, Huiyun Yuan

**Affiliations:** Renji Hospital Shanghai Jiao Tong University School of Medicine, NO.160 Pujian Road, ShangHai, 200127 China

**Keywords:** Hospital nursing care worker, Turnover intention, Related factors

## Abstract

**Background:**

Currently, hospital nursing care workers (hereafter referred to as HNCWs) have become an important part of the healthcare system in China. They exist in nearly all of the public hospitals and in some private hospitals, making up 20 to 30% of the total nursing staff and providing 30 to 40% of basic nursing care for patients. However, many studies have shown that the turnover rate of HNCWs is very high, with average annual rates of 20 to 45%. We conducted this survey to explore their turnover intentions and related factors and present some suggestions to improve their retention rate.

**Methods:**

A total of 514 HNCWs employed at 11 hospitals in Shanghai participated in this study. The inclusion criteria were as follows: (1) being a certified HNCW, (2) having worked as an HNCW for more than 1 year, and (3) volunteering to take part in the survey.

**Results:**

The overall turnover intention of the HNCWs was 41.3%. Influencing factors include education (βeta = 0.201, *P* = 0.000), wages (βeta = − 0.920, *P* = 0.000), management satisfaction, (βeta = − 0.213, *P* = 0.000), satisfaction with wages (βeta = − 0.612, P = 0.000), satisfaction with working hours (βeta = − 0.270, *P* = 0.000), satisfaction with their own work (βeta = − 0.066, *P* = 0.027), work stress (βeta = 0.726, *P* = 0.000), enjoyment of the job (βeta = − 0.141, *P* = 0.000) and hours of sleep (βeta = − 0.046, *P* = 0.037).

**Conclusions:**

Decreasing HNCWs’ turnover intentions and the overall turnover rate is important for improving the quality of healthcare. More attention should be paid to this issue in the enactment of health policy.

## Background

In China, hospital nursing care workers (HNCWs) are those who work in hospitals and provide basic nursing care to patients or disabled elders, including feeding, dressing, bathing, and toileting. Their duties also include observing and reporting changes in patients’ physical and psychosocial status under the instruction of doctors and nurses.

HNCWs as an occupation came into being in China in the 1970s. At first, these workers were hired by individuals to care for sick family members in the hospital, and their salary was paid directly by the hirers. However, at that time, the number of HNCWs was not large, and the quality of service was difficult to guarantee, as nearly all of them were untrained. With the gradual aging of the Chinese population and the increasing incidence of chronic diseases, the demand for HNCWs is surging, and the number of HNCWs has begun to grow since the 1980s, as have the number of HNCW agencies. These companies are responsible for management, training, payroll and other duties. To some extent, these companies relieve the management burden of hospitals. However, there are still many problems and difficulties in HNCW management facing these companies. First, HNCWs are recruited by and sign contracts with these management agencies directly. As most of these positions are not paid well and enjoy relatively low social status in China, many individuals are reluctant to undertake this job. The result is a shortage of HNCW laborers. The agencies cannot meet the urgent societal need for HNCWs. Second, there is no charge standard on HNCWs’ services. The current standard is based on head count each day, which is approximately 150–200 CNY (21.59–28.78 USD) per person per day. If the HNCWs take care of several persons each day, the fee will be reduced to only 60–80 CNY (9.56–12.74 USD) per person per day. Many HNCWs prefer to serve several persons each day because they can obtain more wages, but the quality of service may be lower, which may lead to negative outcomes for the patients. Lastly, the team stability of HNCWs is not solid. On the one hand, HNCWs in China provide 24-h care for patients while their income is the lowest in the hospital; on the other hand, HNCWs are exposed to various harmful substances that may potentially threaten their health, so an increasing number of HNCWs want to leave their work.

HNCWs are similar to the nursing assistants employed in other countries, though with many differences. First, their management departments are different. In other countries, nursing assistants are hired and managed by nursing homes or hospitals directly [1].While in China, HNCWs serve in hospitals, but their labor relations are affiliated with the HNCW agencies. The patients receiving care pay HNCW agencies a fee according to their charge standard. Then, the company will deduct 20% as commission from the total revenue, and the rest will be paid to the HNCWs as salary. Second, compared with nursing assistants in other countries, the training of HNCWs in China is insufficient. Nursing assistants in other countries are also called “certified nursing assistants (CNAs)”. Although their work is often perceived as “unskilled”, they have been systematically trained. In 1987, the federal government of the United States enacted standards for CNA training, requiring CNAs to receive a minimum of 75 h of training within 4 mos of working with patients [2]. The actual number of hours of training that CNAs receive often exceeds the minimum federal and state requirements. In China, there are no standards for HNCW training. HNCWs do not need a prejob qualification certificate, they only need 7–14 days or even much less training time. In fact, many of the HNCWs receive training in less than 7 days because most of them have to pay for the training programs themselves. After passing the tests, they can obtain a certificate that allows them to work. Lastly, the work duties of HNCWs are simpler than those of nursing assistants. Nursing assistants not only assist patients with activities of daily living but also are taught how to measure basic physiological indexes, such as temperature, pulse, respiration, blood pressure, and urine output. However, there is much in common between these positions. They are frequently paid low salaries and receive limited or no benefits. They often face demanding workloads, unsafe working conditions, inadequate training, a lack of respect from others, a lack of control over their jobs, and few opportunities for advancement. All of these factors can result in high turnover.

Currently, HNCWs have become an important part of the healthcare system in China. They exist in nearly all of the public hospitals and some private hospitals, making up 20 to 30% of the total nursing staff and providing 30 to 40% of basic nursing care for patients. However, many studies have shown that the turnover rate of HNCWs is very high, with average annual rates of 20 to 45% [3–8]. In some cities in China, this level of turnover deserves extensive attention. Nursing care is inherently labor intensive [9]. Understanding hospital HNCWs’ turnover intentions and their influencing factors is important and necessary, as they are the keys to improving recruitment and retention strategies [10]. This article discusses the extent, implications, and causes of this turnover and proposes some solutions for addressing the problem [11]. However, no research has yet addressed this important issue.

## Methods

### Study design

A cross-sectional study using convenience sampling was implemented. We invited hospitals that have employed HNCWs for at least 5 yrs to participate in our study and then sent questionnaires to HNCWs in each hospital. The design, protocol, and informed consent procedure of this cross-sectional analysis were approved by the Renji Hospital, which is affiliated with Shanghai Jiao Tong University School of Medicine. All participants included in this study were evaluated by the same independent assessors. The evaluated items included certification, work experience and willingness to participate. The questionnaire was designed to explore the HNCWs’ turnover intentions and related factors, providing an evidence base for managers to help them understand what motivates individuals to work as HNCWs and what contributes to satisfaction with their jobs and to the likelihood of staying in their jobs.

### Sampling and selection criteria

The researchers accessed the list of hospitals employing HNCWs in Shanghai City and contacted the 15 hospitals on the list, inviting them to take part in the study. Only 11 hospitals accepted the invitation. Then, we went to the wards of these hospitals to distribute questionnaires to the HNCWs. The assessors confirmed that these participants met the following inclusion criteria: (1) certified HNCW, (2) a work history as a HNCW for more than 1 yr, and (3) participation in the survey as a volunteer. Ultimately, a total of 600 participants were involved in our survey.

### Instruments

We designed a 23-item questionnaire to measure turnover intention and its drivers. The questionnaire consisted of sections on demographics, time worked as an HNCW, career before becoming an HNCW, wages (CNY), job satisfaction, self-evaluation, work stress, accident insurance, enjoyment of the job, vacation per year, hours of sleep per night, turnover intention, etc. A total of 600 questionnaires were sent to the HNCWs who met the selection criteria, and 541 responded (response rate, 90.2%). Among these questionnaires, 514 were usable (95.9%).

### Statistical analyses

The data was collected in Epidata version 3.1 and exported to SPSS version 20 for analysis. Chi-square and binary logistic regression tests were conducted to determine the turnover intention of the HNCWs and to identify what factors contribute to their leave. First, we point out the turnover intention among different HNCWs with descriptive statistical methods. Second, we perform binary logistic regression tests (dependent variable is “whether he/she wants to leave”; independent variable is different influencing factors). Lastly, we identify the factors behind HNCWs’ leave intentions. In this study, variables that had a *P*-value < 0.05 were considered significantly associated variables. The results can be seen in Table 1.

**Table 1:** Sample characteristics and main themes of the questionnaire (*N* = 514)

Characteristics	% or mean (SD)	Turnover intention (yes)	Turnover intention (no)
Gender		Chi^2^ = 9.16^b^	
Female	83.6%	55.7%	44.3%
Male	16.4%	73.0%	27.0%
Age	49.1 (5.5)	Chi^2^ = 0.55	
< 35	1.2%	62.5%	37.5%
36–45	21.9%	42.9%	57.1%
46–55	69.3%	40.5%	59.5%
> 55	7.6%	39.6%	60.4%
Residence		Chi^2^ = 0.26	
City	1%	35.3%	64.7%
Country	99%	41.5%	58.5%
Education		Chi^2^ = 4.89	
Illiterate	22.1%	48.3%	51.7%
Primary school degree	45.5%	36.6%	63.4%
Junior high school degree	30.2%	43.5%	56.5%
Senior high school degree	2.2%	50.0%	50.0%
Length of time working as an HNCW		Chi^2^ = 36.88^c^	
Less than 5 years	29.5%	69.5%	30.5%
5 to 10 years	26.9%	68.5%	31.5%
More than 10 years	43.6%	58.7%	41.3%
Profession before working as an HNCW		Chi^2^ = 16.50^b^	
No job	4.8%	84.1%	15.9%
Farmer	79.0%	57.4%	42.6%
Enterprise worker	8.6%	53.7%	46.3%
Individual business	2.1%	60.0%	40.0%
Other	5.5%	33.3%	66.7%
Wages (CNY)		Chi^2^ = 10.17^a^	
< 2500	3.7%	63.2%	36.8%
2501–4000	45.2%	52.1%	47.8%
4001–6000	50.9%	48.2%	51.8%
> 6000	0.2%	0%	100%
Job satisfaction (1–5)		F = 35.00^c^	
Management	4.06	3.80	4.43
Wages	3.68	3.50	3.94
Working hours	3.47	3.32	3.69
Self-evaluation (1–5)		F = 24.02^c^	
Satisfaction with their own work	3.98	3.92	4.01
Perceived doctor and nurse satisfaction with their work	3.93	3.76	4.17
Perceived patient satisfaction with their work	3.80	3.61	4.06
Work stress		Chi^2^ = 15.48^c^	
High stress	16.0%	47.1%	52.9%
A little stress	57.3%	34.1%	65.9%
No stress	25.7%	31.2%	68.8%
Accident insurance		Chi^2^ = 3.09	
Yes	86.5%	40.1%	59.9%
No	6.6%	50.0%	50.0%
Not sure	6.8%	50.0%	50.0%
Enjoyment of the job		Chi^2^ = 167.28^c^	
Yes	2.7%	7.1%	92.9%
No	61.0%	30.8%	69.2%
Not sure	36.3%	21.5%	78.8%
Vacation per year		Chi^2^ = 7.90	
No vacation	13.1%	49.3%	50.7%%
1–10 days	25.9%	45.9%	54.1%
11–20 days	50.5%	38.2%	61.8%
More than 21 days	10.5%	32.1%	67.9%
Hours of sleep per day		Chi^2^ = 23.11^c^	
< 4 h	9.6%	40.2%	59.8%
4–6 h	58.1%	33.2%	66.8%
7–8 h	26.5%	30.1%	69.9%
> 8 h	5.8%	26.8%	73.2%
Turnover intention		41.3%	58.7%

^c^*p* < 0.001; ^b^*p* < 0.01; ^a^*p* < 0.05

## Results

### Descriptive statistics

As shown in Table 1, the overall turnover intention of HNCWs is 41.3%, which means that nearly half of them intend to quit their job. We can see the following results from Table 1: Turnover intention varied by “gender” (× 2 = 9.16, *P* < 0.01), “length of time working as an HNCW” (× 2 = 36.88, *P* < 0.001), “profession before working as an HNCW” (× 2 = 16.50, P < 0.01), “wages” (× 2 = 10.17, P < 0.01), “job satisfaction” (F = 35.00, *P* < 0.001), “self-evaluation” (F = 24.02, P < 0.001), “work stress” (× 2 = 15.48, P < 0.001), “enjoyment of the job” (× 2 = 167.28, *P* = 0.001), and “hours of sleep per day” (× 2 = 23.11, P < 0.001).

We can draw the following information from Table 1: Males (73.0%) are more willing to leave than females (55.7%) possibly because males often have more responsibility to support their family, so they are under more pressure at work. With respect to “length of time working as an HNCW”, we can see that those who work as an HNCW for less than 10 years are more willing to leave than those who work as an HNCW for more than 10 years. In addition, what merits our attention is that we found that those with no prior job are more likely to quit working as an HNCW. The reasons behind this finding are worthy of exploration. At the same time, we found that if the wages reached 6000 CNY (nearly 957 USD), no HNCW wanted to leave.

In terms of job satisfaction, HNCWs were more satisfied with management status than wages and working hours. There were significant differences in terms of turnover intention (*P* < 0.001), indicating that the HNCW management agencies’ methods and outcomes affect the HNCWs’ leaving intentions to some extent.

The scores for the items “satisfaction with their own work”, “perceived doctor and nurse satisfaction with their work”, and “perceived patient satisfaction with their work” were 3.98, 3.93, and 3.80, respectively. In addition, the difference was statistically significant for turnover intention (*P* < 0.001). Additionally, 16.0% of participants reported high stress, 57.3% reported little stress, and 25.7% reported no stress; the difference was statistically significant for turnover intention (*P* < 0.001). To the question “Do you like the job?”, 2.7% of them replied “yes”, 61% replied “no”, and 36.3% replied “not sure”. The difference was statistically significant for turnover intention (P < 0.001). We can see that those who did not like the job were more willing to leave. Vacation status per year was as follows: 13.1% of participants had no vacation, 25.9% had only 1–10 days of vacation, 50.5% had 11–20 days of vacation, and 10.5% had more than 21 days of vacation. The difference was not statistically significant for turnover intention. With respect to “hours of sleep per day”, 9.6% of participants slept less than 4 h a day; 58.1%, 4–6 h a day; 26.5%, 7–8 h a day; and 5.8%, more than 8 h a day. This difference in sleep hours was statistically significant for turnover intention (*P* < 0.001). Those who slept less than 6 h a day were more likely to leave.

### Binomial logistic regression model

As shown in Table 2, the binomial logistic regression shows that “education”, “wages”, “management satisfaction”, “satisfaction with wages”, “satisfaction with working hours”, “satisfaction with their own work”, “work stress”, “enjoyment of the job” and “hours of sleep” significantly affect the turnover intention of HNCWs (*P* < 0.01). Of the studied variables, only “education” (Beta = 0.201, *P* < 0.01) and “work stress” (Beta = 0.726, *P* < 0.01) have a positive influence on turnover intention. The higher the HNCWs’ education level is, the more likely they want to leave. Those who experience more work stress are also more likely to leave. Additionally, “wages” (Beta = − 0.920, *P* < 0.01), “management satisfaction” (Beta = − 0.213, P < 0.01), “satisfaction with wages” (Beta = − 0.612, P < 0.01), “satisfaction with working hours” (Beta = − 0.270, P < 0.01), “satisfaction with their own work” (Beta = − 0.066, *P* < 0.05), “enjoyment of the job” (Beta = − 0.141, P < 0.01) and “hours of sleep” (Beta = − 0.046, P < 0.05) have a negative influence on turnover intention. Individuals who have higher wages, higher management satisfaction, and more satisfaction with their wages and working hours are more willing to stay at their work.

**Table 2:** Binomial logistic regression model for turnover intention

Variable	Beta	*T-*value	*P*-value
Gender	0.013	0.592	0.554
Age	0.036	0.859	0.391
Residence	0.013	0.620	0.536
Education	0.201	7.123	0.000^a^
Time worked as an HNCW	−0.013	− 0.582	0.561
Career before becoming an HNCW	0.030	1.413	0.158
Wages	− 0.920	−35.785	0.000^b^
Management satisfaction	−0.213	−7.019	0.000^b^
Satisfaction with wages	−0.612	−23.561	0.000^b^
Satisfaction with working hours	−0.270	−7.317	0.000^b^
Satisfaction with their own work	−0.066	−3.177	0.027^a^
Perceived doctor and nurse satisfaction with their work	−0.011	−0.412	0.680
Perceived patient satisfaction with their work	−0.010	−0.393	0.694
Work stress	0.726	30.092	0.000^b^
Accident insurance	−0.006	− 0.253	0.801
Enjoyment of the job	−0.141	−5.432	0.000^b^
Vacation per year	−0.104	−0.155	0.877
Hours of sleep	−0.046	−2.088	0.037^a^

^b^*p* < 0.01; ^a^*p* < 0.05

## Discussion

High HNCW turnover rates are one of the most important factors contributing to the worldwide shortage of certified nursing assistants, direct care workers, personal care attendants or similar unskilled health workers [12]. They provide most of the direct care received by patients in hospitals or by residents in nursing homes. Similar to certified nursing assistants (CNAs) in other countries, HNCWs make up 60 to 70% of the total nursing staff in nursing homes and provide 80 to 90% of the direct care for nursing home residents [13]. CNAs perform an important role in other countries. However, some studies have indicated that CNA turnover in long-term care (LTC) facilities is a major problem: the turnover rates range from 80 to 100% per year in the United States [2]. Numerous published studies have identified risk factors for high CNA turnover, including low salary, low staff/patient ratios, inadequate orientation, and lack of respect and recognition [14–17]. In China, since HNCWs play an important role in hospitals and long-term care facilities, studies on their psychological health [18], career competency [19], training program [20] and management mode have recently received an increasing amount of attention. However, few studies have concentrated on their turnover intention and related factors. In our study, we found that HNCWs have very high turnover intentions and that 41.3% of HNCWs in Shanghai intend to leave within 1 yr. According to the results of our study, the turnover intention may be driven by the following factors.

First, educational background is an important influencing factor of HNCWs’ turnover intention. One result of our survey shows that those who are illiterate and obtain a senior high school degree are more likely to leave their job. The reason for this finding may include two aspects. On the one hand, the work duties of HNCWs include basic medical services, such as “observing and reporting changes in patients’ physical and psychosocial status”. Those who are illiterate may not be able to accomplish this work, so many of them consider leaving. On the other hand, the ones with a higher education degree may prefer to choose more a challenging job, so they have a willingness to leave.

Second, HNCWs’ wages are lower than those of other similar health workers. As we can draw from the descriptive statistics, most HNCWs earn less than 2500 CNY (nearly 400USD) every month, and although their wages are above the lowest wage standard in Shanghai (2190CNY, nearly 351USD), these wages cannot meet the needs of their lives. At the same time, their wages are lower than those of other healthcare workers, such as nurses and doctors, but their work burden is the heaviest among them. From the binomial logistic regression model, we can see that the wages are one of the factors influencing HNCWs’ turnover intentions. Many HNCWs are not satisfied with their wages and have to seek a higher-salary job.

Third, the management mode may influence HNCWs’ willingness to leave. In our survey, we find that “management satisfaction” is one of the factors influencing their intention to leave. As the HNCW is a newly born profession in China, the laws and regulations governing it are not complete. There are no standards on their rights and obligations, work contents, payment level, career security and rights protections. Management modes are also different among companies. It is common that some HNCWs leave one company and go to another one, which can increase the costs of recruiting and training them.

Lastly, work stress and long working hours contribute to the high turnover intentions among HNCWs. In our survey, we found that 16% of HNCWs feel that their work stress is “high”. At the same time, 58.1% of them sleep less than 6 h per night. These two factors have an influence on HNCWs’ turnover intentions.

Above all, low wages, high work stress, little sleep, and other factors, such as low social status and limited resources, result in the high turnover intentions of HNCWs.

The fact that a large number of HNCWs want to leave may have a negative influence on the quality of care of facilities. We have some recommendations.:First, we should improve the payment mechanism, unify payment standards, and increase their wages. Second, there is a need to offer them professional development opportunities and reasonable career pathways. For example, we can provide chances for those who have worked for more than 10 years to pursue further education and allow them to take part in the nurse qualification exam. Another recommendation is to offer HNCWs short training programs in management, leadership, equal opportunity and communication skills. Third, a strategy that may also assist in managing heavy workloads and enhancing HNCWs’ job satisfaction is the extension of HNCWs’ roles to involve simple nursing tasks under the direction of a registered nurse, as is done in many other countries. Fourth, to curb favoritism, the hospital system needs to be clear about equity and fairness for all HNCWs. Consultation with management expertise might be helpful, as they can explain why certain behaviors are not consistent with Chinese hospitals’ religious management model. Finally, equal-opportunity policies based on merit would facilitate the retention of HNCWs from diverse cultural backgrounds. It is particularly important to introduce innovative and locally responsive systems, such as native-language classes for those with a first language other than that of the city in question, seminars to improve cultural awareness and cross-cultural communication among HNCWs, spirituality workshops to help HNCWs cope with job stress, and appropriate job responsibilities.

### Limitations

This study has limitations. First, in this study, we used convenience sampling. This technique may affect the representativeness of the sample to some extent. Taking this limitation into consideration, we increased the number of samples, thus strengthening the assessment towards the sample. However, random sampling methods should be used in future studies. Second, there is the possibility of response bias, as some of the HNCWs are illiterate. Thus, there may be some deviation in their understanding of the questions. To solve this problem, our investigators would explain the questions to the illiterate HNCWs during investigation. Finally, this study was conducted in only 11 hospitals in a single city. Caution should be exercised in making generalizations across different hospitals and countries.

## Conclusion

HNCWs’ turnover intentions have a significant influence on work enthusiasm, which is crucial to the improvement of nursing and medical care in hospitals. The results of the survey are striking, and the suggestions we make can decrease HNCWs’ turnover intentions and rates, thus improving the quality of healthcare, and are useful for other countries that have similar healthcare workers. More attention should be paid to this problem in the enactment of future health policies.

## Abbreviations

CNA: Certified nursing assistant
HNCW: Hospital nursing care worker
LTC: Long term care
